# Three-Dimensional Bioprinting of Functional Skeletal Muscle Tissue Using Gelatin Methacryloyl-Alginate Bioinks

**DOI:** 10.3390/mi10100679

**Published:** 2019-10-09

**Authors:** Rasoul Seyedmahmoud, Betül Çelebi-Saltik, Natan Barros, Rohollah Nasiri, Ethan Banton, Amir Shamloo, Nureddin Ashammakhi, Mehmet Remzi Dokmeci, Samad Ahadian

**Affiliations:** 1 Department of Bioengineering, Samueli School of Engineering, University of California-Los Angeles, Los Angeles, CA 90095, USA; Rasoul.Seyedmahmood@gmail.com (R.S.); barrosnr@g.ucla.edu (N.B.); rhnasiri90@gmail.com (R.N.);; 2 Center for Minimally Invasive Therapeutics (C-MIT), University of California-Los Angeles, Los Angeles, CA 90095, USA; dokmeci1@gmail.com; 3 College of Engineering, University of Missouri, Columbia, MI 65211, USA; 4 Department of Stem Cell Sciences, Graduate School of Health Sciences, Hacettepe University, Ankara 06100, Turkey; 5 Department of Mechanical Engineering, Sharif University of Technology, Tehran 11365-11155, Iran; shamloo@sharif.edu; 6 Department of Radiological Sciences, David Geffen School of Medicine, University of California-Los Angeles, Los Angeles, CA 90095, USA

**Keywords:** muscle tissue engineering, GelMA-alginate bioink, 3D bioprinting, oxygen-generating bioink

## Abstract

Skeletal muscle tissue engineering aims to fabricate tissue constructs to replace or restore diseased or injured skeletal muscle tissues in the body. Several biomaterials and microscale technologies have been used in muscle tissue engineering. However, it is still challenging to mimic the function and structure of the native muscle tissues. Three-dimensional (3D) bioprinting is a powerful tool to mimic the hierarchical structure of native tissues. Here, 3D bioprinting was used to fabricate tissue constructs using gelatin methacryloyl (GelMA)-alginate bioinks. Mechanical and rheological properties of GelMA-alginate hydrogels were characterized. C2C12 myoblasts at the density 8 × 10^6^ cells/mL were used as the cell model. The effects of alginate concentration (0, 6, and 8% (w/v)) and crosslinking mechanism (UV crosslinking or ionic crosslinking with UV crosslinking) on printability, cell viability, proliferation, and differentiation of bioinks were studied. The results showed that 10% (w/v) GelMA-8% (w/v) alginate crosslinked using UV light and 0.1 M CaCl_2_ provided the optimum niche to induce muscle tissue formation compared to other hydrogel compositions. Furthermore, metabolic activity of cells in GelMA bioinks was improved by addition of oxygen-generating particles to the bioinks. It is hoped that such bioprinted muscle tissues may find wide applications in drug screening and tissue regeneration.

## 1. Introduction

Skeletal muscle tissues aid the body in support, locomotion, and even in the regulation of metabolism. Muscle tissue is comprised of muscle fibers bundled together by the perimysium, which are then axially aligned and grouped together by the epimysium to form skeletal muscle tissues [1]. These muscle fibers are formed through the fusion of myoblast cells into myotubes, elongated and cylindrically shaped multi-nucleated cells [2]. Skeletal muscle tissue, together with tendon tissue, are involved in the effort transmission to bone tissue leading to the body movement [3]. Skeletal muscle tissue engineering aims to develop functional skeletal muscle tissue constructs [4,5]. These constructs can be used to replace damaged muscle tissues, act as *in vitro* models for understanding the growth and development mechanisms of the muscular system, and for testing different drugs for treatment of muscular diseases and injuries [6,7]. Mimicking highly packed and organized cellular organization of the native muscle tissues, using natural or synthetic scaffolds and microscale technologies, is crucial for successful engineering of skeletal muscle tissues [8,9].

Three-dimensional (3D) bioprinting has emerged as a powerful microscale technology for tissue biofabrication, offering the ability to customize shape, material, and structure of tissue scaffolds [10,11]. Bioprinting has also allowed for the incorporation of cells and soluble factors during printing process [12]. This process has recently become popular in fabricating different tissue and organ constructs due to its ability to mimic the hierarchical structure of the native tissues [13,14]. In particular, 3D bioprinting has been used in skeletal muscle tissue engineering. For example, Kim et al. created 3D printed muscle tissues using human primary muscle progenitor cells [15]. The printed cells were able to form dense and highly aligned muscle fibers. Following the implantation in rat models, major functionality of tibialis anterior defect was restored. In another study, aligned muscle fibers of C2C12 cells in pluronic/alginate blend bioinks were fabricated using direct-write bioprinting [16]. The fabricated muscle fibers showed higher performance compared to two-dimensional cultured cells. Microfluidic-based bioprinting was used to print myotubes using polyethylene glycol-fibrinogen bioink [17]. The muscle myofibers showed sarcomeric organization and enhanced muscle regeneration in immunocompromised mice models. More recently, Testa et al. used the same strategy and printed human muscle cells derived from perivascular and pericyte stem cells to treat sphincter muscle defects [18]. These approaches used bioinks with limited tunability in physicochemical and biological properties. In particular, the scaffolds should mimic mechanical properties of the extracellular matrix of the native muscle tissues, while those properties should be compatible with bioprinting method in terms of printability and preserving cell viability and function.

Cell-laden hydrogels have been widely used as bioinks to fabricate different tissue constructs [19]. In particular, gelatin methacryloyl (GelMA) is a known hydrogel with tunable properties that can mimic the native muscle tissue environment [4]. We have done several studies showing the suitability of GelMA hydrogel and its composites with different nanomaterials for muscle tissue engineering [20,21]. However, GelMA itself may not have enough viscosity for bioprinting procedures. Excessive amounts of alginate can increase the strength of GelMA bioinks, which reduces the bioactivity of the composite ink [22]. Therefore, alginate concentration within GelMA bioinks should be optimized to achieve the desired viscosity for bioprinting, and to obtain high cellular viability and function of skeletal muscles cells.

In the present study, two different methods of crosslinking GelMA/alginate inks were used: UV light (for GelMA hydrogel) and combined UV light with ionic crosslinking (for GelMA and alginate hydrogels). Viscosity and injectability of the inks were evaluated using rheology and muscle myotube formation was measured using anti-Desmin antibody immunostaining. Cell viability and metabolic activity of the mentioned cell-laden bioinks were investigated. Finally, by adding oxygen-releasing particles (i.e., calcium peroxide (CPO)) to the GelMA bioink, the optimal percentage of the CPO was used to enhance the viability and metabolic activity of C2C12 cells.

## 2. Materials and Methods

All reagents and cell culture media were purchased from Gibco (Grand Island, NY, USA). C2C12 mouse model cells were purchased from the American Type Culture Collection (Manassas, VA, USA). Alexa Flour 488 and 568 antibodies were purchased from Thermo Fischer Scientific (Waltham, MA, USA). Laboratory chemicals and reagents were purchased from Sigma-Aldrich (St. Louis, MO, USA) unless specified otherwise. Penicillin/Streptomycin (P/S) was purchased from Invitrogen (New York, NY, USA).

### 2.1. Gelatin Methacryloyl (GelMA) Synthesis

GelMA solution was prepared by following previous instructions [23]. In short, 10 g of gelatin type A (porcine skin, Sigma-Aldrich, St. Louis, MO, USA) was dissolved in 100 mL of Dulbecco’s Phosphate-Buffered Saline (DPBS) (Gibco) at 50 °C before adding 8 mL of methacrylic anhydride, in which acrylation reaction carried out for 1 h under constant stirring. Dilution of the reaction with 200 mL of warm (40 °C) DPBS terminated excess methacrylation. Resulting mixture was dialyzed for 1 week with warm distilled water (40 °C) and 12–14 kDa cut-off dialysis tube before lyophilization for 1 week.

### 2.2. Three-Dimensional Bioprinting Procedure

Bioinks consisted of 10% (w/v) GelMA, alginate (6 or 8% (w/v)), 0.5% (w/v) photoinitiator (2-hydroxy-4′-(2-hydroxyethoxy)-2-methylpropiophenone), and muscle cells. Crosslinking solution contained a concentration of 0.1 M CaCl_2_. The bioinks and the crosslinking solution were prepared in DPBS and distilled water, respectively, and kept at 37 °C before use. The bioprinting was done using Inkredible 3D Bioprinter (CELLINK, Boston, MA, USA). Parameters of bioprinting process are summarized in Table 1.

### 2.3. Mechanical and Rheological Properties of Hydrogels

Mechanical properties of bulk GelMA-alginate hydrogels were quantified using unconfined compressive tests (Instron 5542). The pre-polymer solutions were poured in glass molds (1 mm height) and placed at 4 °C for 2 h to induce the thermal gelation of GelMA. Cylindrical discs (1 mm height and 5 mm diameter) were cut using a 5 mm biopsy punch, soaked in 0.1 M CaCl_2_ solution for 2 min, then exposed to UV light (800 mW/cm^2^ for 30 s) using Omnicure, S2000 (Excelitas Technologies, Waltham, MA, USA), washed with DPBS, and placed in cell culture media in an incubator to obtain the swelling equilibrium. After 24 h of incubation at 37 °C mechanical testing was performed. The rheological properties of hydrogels dissolved in DPBS were obtained using a rotational rheometer (Anton Paar RHEOPLUS-32, plate-cone geometry). The solutions were placed under a stirrer at room temperature for 2 h before the experiment. The experiment was done in a closed environment at 25 °C to avoid evaporation. 

### 2.4. Cell Culture and Cell Viability Assay

Mouse-derived C2C12 myoblast cells were cultured in 10% fetal bovine serum, 1% P/S, and 20 mM 4-(2-hydroxyethyl)-1-piperazineethanesulfonic acid supplemented with Dulbecco’s Modified Eagle Media (DMEM) at 37 °C and 5% CO_2_. Cell trypsinization was done using trypsin/ethylenediaminetetraacetic acid after they reached ~80% confluency. GelMA/alginate gels with and without CaCl_2_ treatment were mixed with mouse model cells (cell density = 8 × 10^6^ cells/mL) before printing. To stabilize 3D cell-laden gels after printing, samples were subjected to UV light for 30 s. Culturing was carried out for 12 days in C2C12 culture medium. Live/dead staining assay including 0.5 µL calcein AM and 2 µL ethidium homodimer per 1 mL DPBS (Thermo Fischer Scientific, Waltham, MA, USA) was conducted to quantify C2C12 cell viability within printed hydrogels.

### 2.5. Quantification of Cell Metabolic Activity 

Effect of alginate (0%, 6%, 8%) on cell viability in GelMA bioinks was assessed using PrestoBlue assay (Thermo Fischer Scientific, Waltham, MA, USA). Briefly, 3D constructs were washed with DPBS. Then, 10% PrestoBlue reagent in culture media was added to the constructs and incubated for 2 h at 37 °C. 100 µL of media was transferred into 96-well plates and the absorption of media at 450 nm was measured using a microplate reader (Synergy™ HTX Multi-Mode Microplate Reader, BioTek Winooski, Winooski, VT, USA).

### 2.6. Immunofluorescent Staining

C2C12 cells were grown in culture medium for 5 days before transferring to differentiation medium (High-glucose DMEM supplemented with 2% horse serum and 1% P/S) for 7 days. The myotubes were treated with 3.7% paraformaldehyde for 20 min, then permeabilized using 0.3% triton X-100 in 5 min. Samples were blocked from binding to nonspecific antibodies using 5% rabbit serum in DPBS for 30 min, then washed with DPBS for 5 min. Primary antibody incubation of monoclonal antibodies was carried out to achieve optimal concentrations in a humidified chamber at 4 °C (1/100 dilution of anti-Desmin (Abcam, Cambridge, UK)) overnight. Sections were washed with 0.05% tween solution and incubated with Alexa Fluor 488-conjugated rabbit anti-mouse IgG containing 5% rabbit serum. 4′,6-diamidino-2-phenylindole was used to perform counterstaining of cell nuclei.

### 2.7. Statistical Analysis

Statistical analysis was performed using one-way ANOVA and student’s *t*-test as implemented in Origin Software (OriginLab Corporation, Northampton, MA, USA). Values of *p* < 0.05 were considered to be statistically significant.

## 3. Results

**Figure 1:**
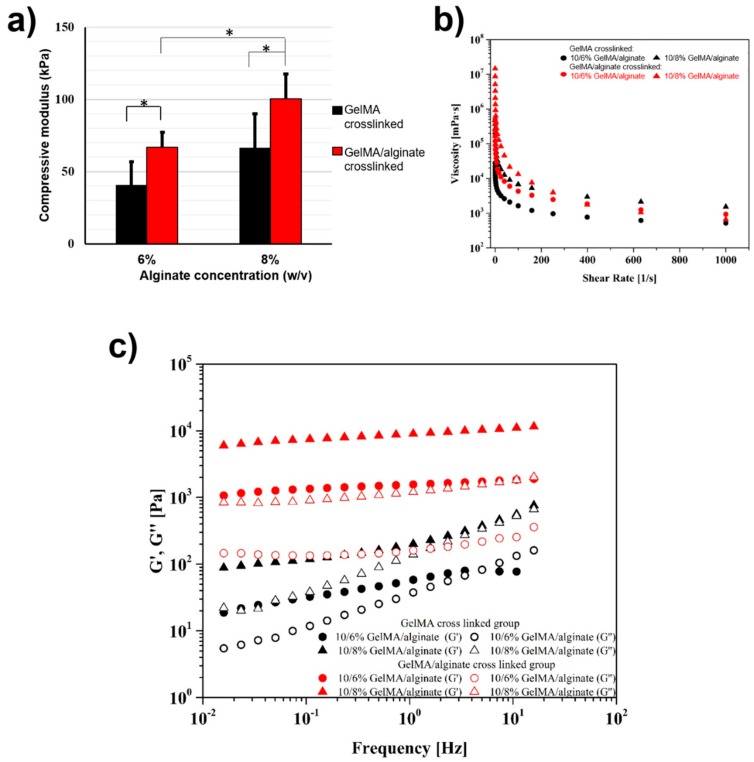
Mechanical and rheological properties of GelMA and GelMA-alginate hydrogels. (**a**) Compressive moduli of 10% (w/v) GelMA with two different alginate concentrations (6% and 8% (w/v)). Different colors indicate different crosslinking methods. Black bars are representative of UV crosslinking method and red bars indicate soaked sample into 0.1 M CaCl_2_ bath for 2 min prior to the UV crosslinking (n = 6; * *p* < 0.05). (**b**) Rheological properties of GelMA and GelMA-alginate hydrogels. Viscosity as a function of shear rate at 27 °C for different hydrogel samples. (**c**) Storage and loss moduli for underlying hydrogels.

Three-dimensional and functional muscle tissue constructs with optimized mechanical and biological properties are required in tissue regeneration and drug screening applications. Three-dimensional bioprinting has evolved as a powerful technology to create biomimetic muscle tissues through the precise positioning of cell-laden biomaterials. However, it is still needed to develop bioinks having biological and mechanical properties suitable for muscle cell growth and differentiation. Here, we proposed GelMA-alginate hydrogels to make musculogenic bioinks. The fabrication mechanism of cell-laden hydrogels was based on using two independent crosslinking processes (i.e., UV crosslinking of GelMA or ionic crosslinking of alginate followed by GelMA crosslinking). The ionic crosslinking provided a temporary stability of printed structures, while the following UV crosslinking enhanced the mechanical stability of the constructs. Figure 1a shows the compression modulus of underlying hydrogels. As expected, the alginate concentration and UV crosslinking had positive effects on the mechanical properties of GelMA-alginate hydrogels, and significantly increased the compressive modulus of GelMA-alginate hydrogels. The viscosity of GelMA-alginate hydrogels increased as the alginate concentration was increased (Figure 1b). The dual crosslinking (ionic and UV crosslinking) of GelMA-alginate gels caused further increases in gel viscosity compared to the ionic crosslinking of the gels (Figure 1b). Rheological data for the hydrogels also confirmed the injectability of all underlying hydrogels and their suitability for bioprinting process (Figure 1c). 

**Figure 2:**
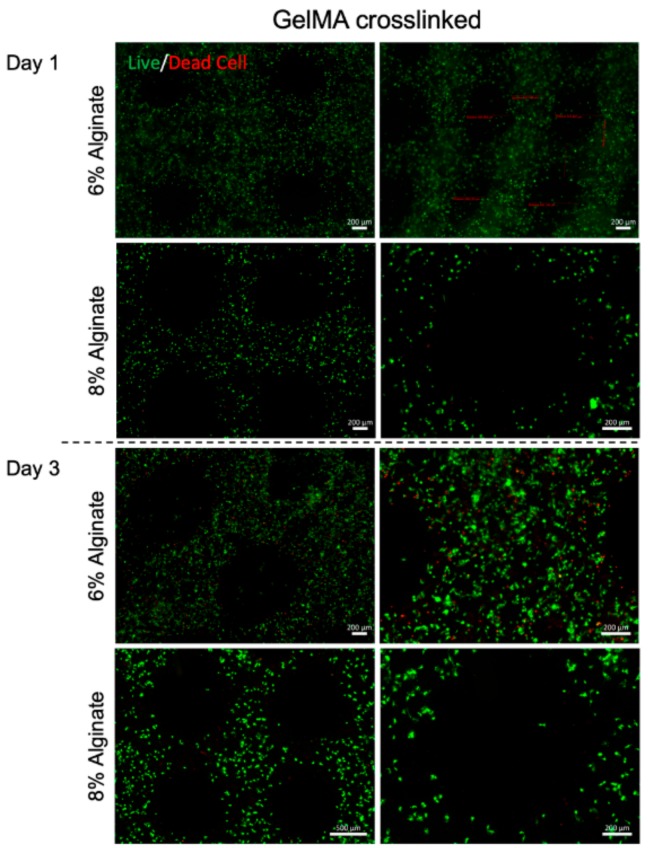
Live/Dead cell staining of C2C12 printed in GelMA hydrogel with alginate concentrations 6% and 8% (w/v) crosslinked with UV light. Images were taken on days 1 and 3 after bioprinting. Green color represents healthy cells and red color represents dead cells.

**Figure 3:**
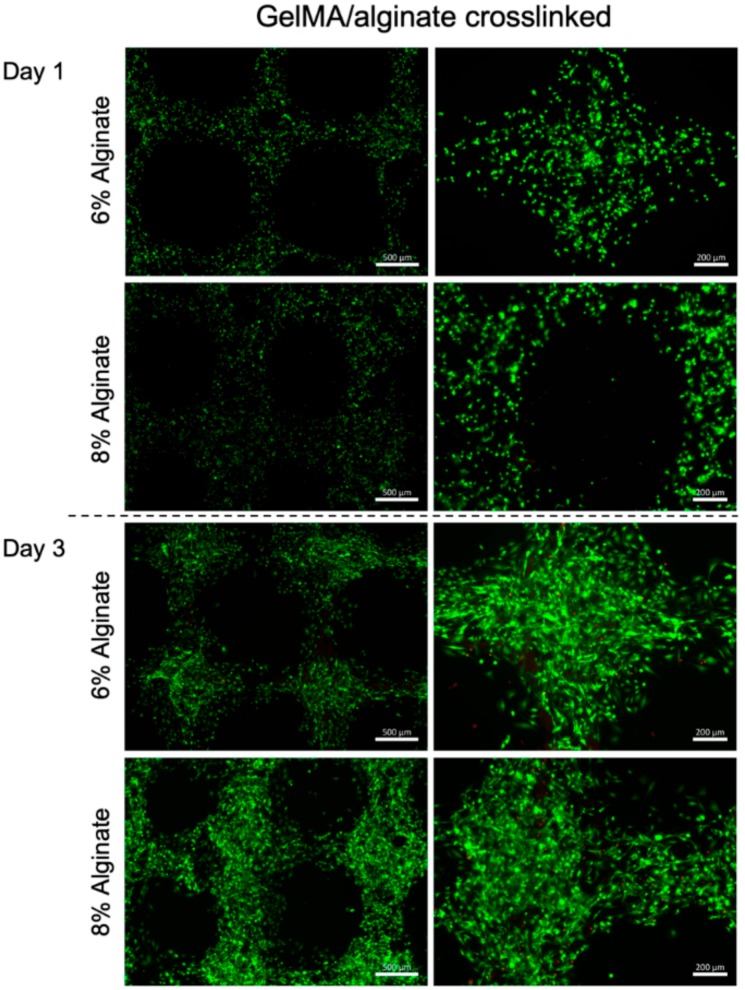
Live/Dead cell staining of C2C12 printed in GelMA hydrogel with alginate concentrations 6% and 8% (w/v) crosslinked with UV light and CaCl_2_ crosslinking. Images were taken on days 1 and 3 after bioprinting. Green color represents healthy cells and red color represents dead cells.

**Figure 4:**
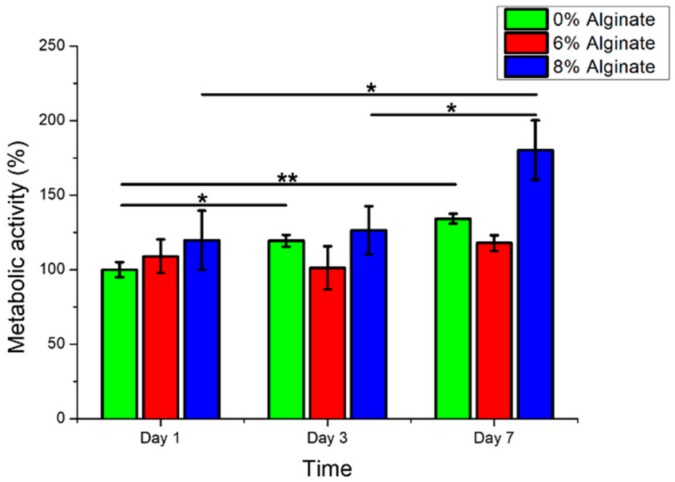
Metabolic activity assay from C2C12 cells in GelMA hydrogel with alginate concentrations 6% and 8% (w/v) crosslinked with UV and CaCl_2_ over time (* *p* < 0.05 and ** *p* < 0.01).

Bioprinted muscle cells were stained as live and dead cells after 1 and 3 days of culture. The results showed that the bioprinting process did not affect the cell viability, as most of the cells were alive in the bioprinted constructs after 1 day of culture (Figure 2). Moreover, the viable cells within dually crosslinked GelMA-alginate bioinks had significantly higher growth compared to the cells in UV crosslinked GelMA-alginate bioinks (Figure 3). We believe that the mechanical properties of scaffolds affected the cell spreading and proliferation. In this regard, dually crosslinked GelMA-8% (w/v) alginate bioinks provided a more favorable mechanical microenvironment for C2C12 cell proliferation compared to other bioinks. We further confirmed the cell viability results with metabolic activity assay (Figure 4), in which cells in dually crosslinked GelMA-8% (w/v) alginate bioinks showed significant improvement in metabolic activity over 7 days of culture (~50%) compared to cells in dually crosslinked GelMA-6% (w/v) alginate. Cells in dually crosslinked GelMA-6% (w/v) alginate did not show significant improvement in the metabolic activity, while cells in GelMA bioinks improved their metabolic activity ~25% over 7 days of culture.

**Figure 5:**
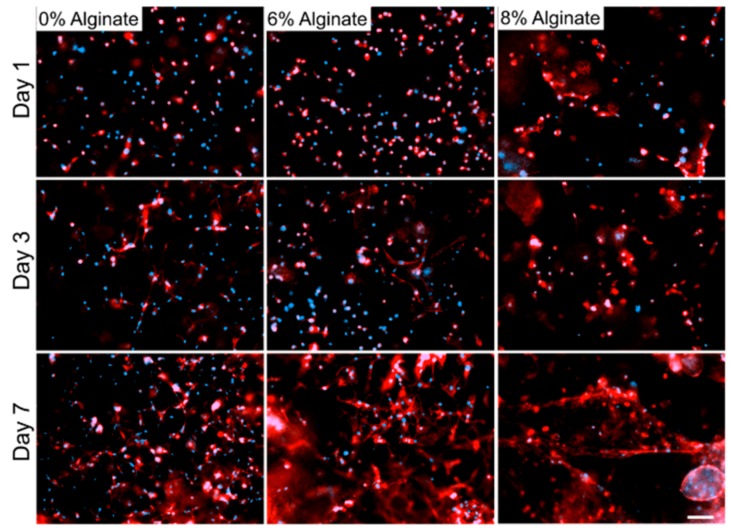
DAPI and F-actin staining of C2C12 in GelMA hydrogel with alginate concentrations 6% and 8% (w/v) crosslinked with UV and CaCl_2_. Images were taken on days 1, 3, and 7. Blue color represents the cell nucleus and red color represents the cell cytoskeletal by the F-actin microfilaments. Scale bar denotes 200 µm.

C2C12 myoblasts cultured in GelMA and GelMA-alginate hydrogels started to spread after 3 days post-printing (Figure 5). In this set of experiments, all GelMA-alginate samples were dually crosslinked with UV and CaCl_2_ as we obtained higher cellular viability and proliferation for them compared to other samples. After 7 days, the cell spreading increased considerably, especially for the samples with 6% (w/v) alginate. However, more organized structures were formed by the cells in GelMA-8% (w/v) alginate as a result of the cell spreading. Increased capacity in muscle cell spreading inside bioinks provides better opportunity for cell-cell connection, increased cell viability, and proliferation. Furthermore, myoblasts would have a higher chance to fuse together and form muscle myotubes during the differentiation process. Here, a relatively low cell density (8 × 10^6^ cells/mL) was used to have a better visualization of cell spreading. However, higher cell density will definitely help in myoblast differentiation to myotubes. Based on these results, a considerable difference in cell viability among the samples can also be explained by comparing the cell spreading results obtained for GelMA bioinks samples in contrast to other samples, as cell spreading and communication are key steps for initiating cell proliferation.

**Figure 6:**
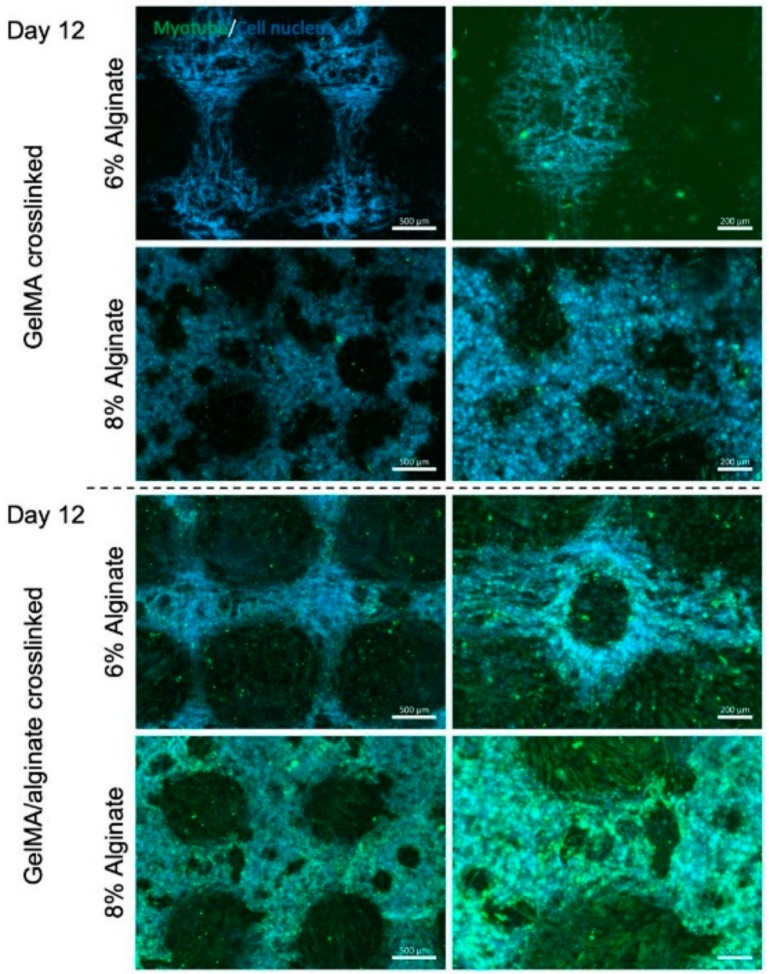
Anti-Desmin staining of C2C12 myotubes in GelMA hydrogel with alginate concentrations 6% and 8% (w/v) crosslinked with either UV light or UV/CaCl_2_ crosslinking. Images were taken on day 12 after bioprinting. Staining was performed to quantify myotube formation, which is marked in green. Imaging shows increased myotube formation upon alginate crosslinking with CaCl_2_.

Staining of cell nuclei and myotubes were further performed to evaluate the effects of crosslinking mechanism of bioinks and alginate concentration on myotube formation (Figure 6). The results showed that dually crosslinked GelMA-alginate bioinks with 8% (w/v) alginate promoted the myotube formation compared to other bioinks. This result is consistent with the previous report that scaffolds for myoblast culture require a compression modulus of at least 200 kPa to tolerate cell-induced deformation in the differentiation process [24,25]. High mechanical strength of dually crosslinked GelMA-8% (w/v) alginate bioinks supported cell-cell contact and thereby cell-cell fusion during the differentiation process more as compared to other bioinks. As a result, the myoblast differentiation was more profound in these hydrogels compared to other underlying hydrogels.

**Figure 7:**
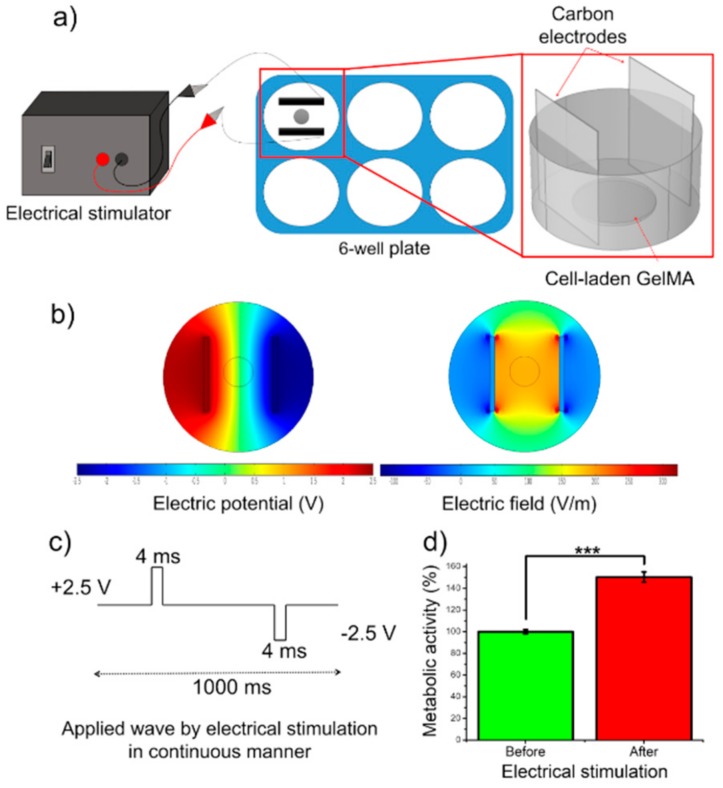
Effect of electrical stimulation on C2C12-laden GelMA bioinks. (**a**) Schematic of electrical stimulation setup. (**b**) Numerical simulation of applied electrical pulse. (**c**) Wave form used to stimulate cells. (**d**) Metabolic activity of C2C12 cells in GelMA bioinks before and after electrical stimulation (*** *p* < 0.001).

Electrical stimulation is an efficient way to increase the maturation of muscle myotubes and to enhance their alignment in the direction of electric field [21]. In this study, a two-channel stimulator (AFG1000 Series Arbitrary/Function Generator, Tektronix, Beaverton, OR, USA) was used to apply square-wave and biphasic pulses to cell-laden GelMA constructs through C-Dish carbon electrodes (IonOptix, Milton, MA, USA). The schematic of stimulation setup is shown in Figure 7a. The cell-laden constructs were placed in the middle of the C-Dish where the carbon electrodes were 2.3 cm distance from each other. We did not apply the electrical stimulation to GelMA-alginate constructs as it was impossible to visually observe and record the tissue beating. The stimulation parameters were voltage amplitude, 2.5 V; frequency, 1 Hz; and duration, 4 ms. Finite element analysis was performed by COMSOL Multiphysics^®^ Modeling Software to quantify the electric field generated by the electrical stimulation around the tissue constructs. The numerical simulation results showed a strong and localized electric field generated between the electrodes, and the electric potential was linearly distributed between the electrodes (Figure 7b). Upon applying Vpp of 5 V using the stimulation setup, an electric field of 2.2 V/cm was obtained. Furthermore, the electric field strength outside the electrode coverage area decreased to a negligible value as compared to the field strength between the electrodes. Metabolic activity of cell-laden scaffolds was assessed by using PrestoBlue reagent before and after applying the electrical stimulation (Figure 7d). A significant increase in the metabolic activity of the cells was observed after two days of stimulation. This result is in agreement with our previous work in which we showed that a minimum of 1 V/cm stimulation for one day of culture is required to increase the functionality of engineered muscle tissues [26].

Skeletal muscle tissues have high metabolic activity and need high amounts of oxygen and nutrients for proper function. Therefore, it was assumed that supplementing bioinks with an oxygen generating source would help to generate functional skeletal muscle tissue *in vitro* particularly for thick tissue constructs. CPO is one of the most common oxygen-generating biomaterials that can be used in biomedical applications [27]. Two steps are needed for decomposition of CPO to generate oxygen. First, the reaction of CPO with water produces hydrogen peroxide (H_2_O_2_) and Ca(OH)_2_ (as shown in Equation (1)). Second, the produced H_2_O_2_ decomposes to water and O_2_ as shown in Equation (2) [28]. The produced H_2_O_2_ can be toxic to cells. Therefore, a catalase should be used to spontaneously decompose H_2_O_2_ and produce oxygen to maintain the cell viability [29].

CaO_2_ (s) + 2H_2_O→Ca(OH)_2_ (s) + H_2_O_2_ (1)

2H_2_O_2_→O_2_ + 2H_2_O (2)

**Figure 8:**
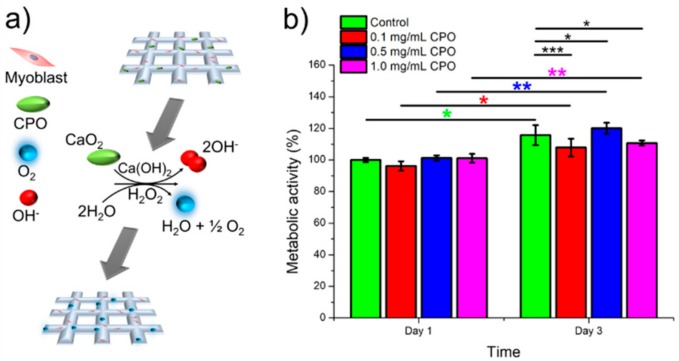
Metabolic activity of C2C12 cells in GelMA bioinks with different CPO concentrations (0.1, 0.5, and 1.0 mg/mL) over time (* *p* < 0.05, ** *p* < 0.01, and *** *p* < 0.001).

**Figure 9:**
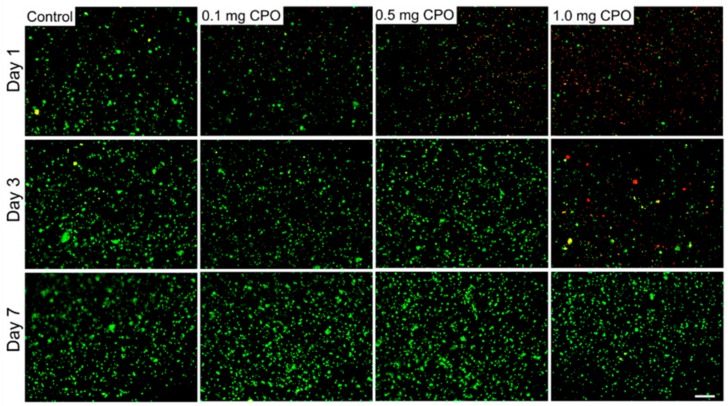
Live/Dead cell staining of C2C12 in GelMA bioinks with different CPO concentrations (0.1, 0.5, and 1.0 mg/mL) crosslinked with UV light. Images were taken on days 1, 3 and 7. Green color represents healthy cells and red color represents dead cells. Scale bar denotes 200 µm.

In order to study the effect of oxygen release kinetics on the viability and function of C2C12 myoblasts in GelMA bioinks, different concentrations of CPO (0.1, 0.5, and 1.0 mg/mL) and catalase (obtained from bovine liver, 100 µg/mL) were added to the GelMA bioinks. Figure 8a shows the schematic for the generation of O_2_ inside the cell-laden GelMA scaffolds. Oxygen release was monitored over 3 days of culture. The metabolic assay results showed that 0.5 mg/mL is optimal concentration for achieving the highest cell survival (Figure 8b). The CPO had negative effect on the cell viability for CPO concentrations above 0.5 mg/mL. This result was confirmed by analyzing live and dead cells for all underlying CPO concentrations as shown in Figure 9. The number of dead cells was significantly increased for 1.0 mg/mL CPO compared to that of 0.5 mg/mL CPO. We did not add CPO to the GelMA-alginate bioinks as we noticed that the calcium chloride ions generated by the CPO caused the physical crosslinking of the alginate. As a result of highly dense bioinks, the cells could not survive for more than one day of culture (data not shown). Therefore, the CPO was added only to GelMA bioinks. These results indicate that oxygen-releasing ingredients in tissue constructs can improve cell viability and proliferation, and it can be a promising method to improve the tissue function, particularly in hypoxic conditions, such as myocardial infarction [30]. The addition of O_2_ generating source to bioinks can open up new avenues in the field of tissue engineering. It is hypothesized that providing O_2_ in bioinks leads to improvement in cell survival and functionality during and after the bioprinting process, which can also be reflected on the subsequent function of the 3D printed constructs *in vitro* and *in vivo*.

## 4. Conclusions

Bioprinted tissue constructs have shown great promise in mimicking the structure and function of the native tissue and organs. However, it is still required to synthesize biomimetic bioinks with tunable physicochemical properties for different tissue engineering applications. Here, we proposed the use of cell-laden GelMA-alginate as the bioink for skeletal muscle tissue fabrication. Mechanical, rheological, and biological properties of these bioinks were assessed as a function of alginate concentration and crosslinking method. The results showed that 8% (w/v) alginate with 10% (w/v) GelMA dually crosslinked with UV and CaCl_2_ can provide the optimal niche for muscle tissue formation. Furthermore, metabolic activity of cells in GelMA bioinks can be improved by the addition of oxygen-generating source to the bioinks. Such bioprinted muscle tissues may find wide applications in drug screening *in vitro* and tissue regeneration *in vivo*.

## Figures and Tables

**Table 1:** Parameters for bioprinting cell-laden GelMA-alginate bioinks.

Fixed Parameters	Value	Note
GelMA percentage (w/v)	10	-
Printing speed (mm/s)	60	-
Printing temperature (°C)	21	-
**Variable Parameters**	-	-
Alginate percentage (w/v)	6–8	-
Post crosslinking mechanism ^a^	UV light 30 s	0.1 M CaCl_2_ bath (2 min) followed by exposing to UV light 30 s

^a^ Based on the crosslinking mechanism proposed in this study as (i) GelMA crosslinked and (ii) GelMA/alginate crosslinked groups.
